# Chewing Gum for Intestinal Function Recovery after Colorectal Cancer Surgery: A Systematic Review and Meta-Analysis

**DOI:** 10.1155/2017/3087904

**Published:** 2017-10-08

**Authors:** Binbin Mei, Wenting Wang, Feifei Cui, Zunjia Wen, Meifen Shen

**Affiliations:** ^1^Department of Neurosurgery, The First Affiliated Hospital of Soochow University & School of Nursing Soochow University, Suzhou 215006, China; ^2^Department of Intensive Care Unit, The First Affiliated Hospital of Soochow University, Suzhou 215006, China; ^3^Nanjing Children's Hospital, Nanjing 210000, China; ^4^Department of Neurosurgery, The First Affiliated Hospital of Soochow University, Suzhou 215006, China

## Abstract

**Background:**

This meta-analysis was performed to assess the efficacy and safety of chewing gum in intestinal function recovery after colorectal cancer surgery.

**Methods:**

A systematic search was conducted in PubMed, Embase, Science Direct, and Cochrane library for relevant randomized controlled trials (RCTs) published until April 2017. Summary risk ratios or weighted mean differences with 95% confidence intervals were used for continuous and dichotomous outcomes, respectively.

**Results:**

17 RCTs with a total number of 1845 patients were included. Gum chewing following colorectal cancer surgery significantly reduced the time to first passage of flatus (WMD −0.55; 95% CI −0.94 to −0.16; *P* = 0.006), first bowel movement (WMD −0.60; 95% CI −0.87 to −0.33; *P* < 0.0001), start feeding (WMD −1.32; 95% CI −2.18 to −0.46; *P* = 0.003), and the length of postoperative hospital stay (WMD −0.88; 95% CI −1.59 to −0.17; *P* = 0.01), but no obvious differences were found in postoperative nausea, vomiting, abdominal distention, pneumonia, and mortality, which were consistent with the findings of intention to treat analysis.

**Conclusions:**

Chewing gum could accelerate the recovery of intestinal function after colorectal cancer surgery. However, it confers no advantage in postoperative clinical complications. Further large-scale and high-quality RCTs should be conducted to confirm these results.

## 1. Introduction

Colorectal cancer is one of the most common tumors, which is associated with multiple risk factors and accounts for approximately 10% of cancer-related mortality in western countries [1, 2]. With increased colorectal cancer incidence, the number of colorectal cancer surgery has also elevated dramatically. Laparoscopic or open colorectal resections, as the main surgical approach for colorectal cancer, may also result in many serious complications like postoperative ileus, nausea, and vomiting, which could lead to significant postoperative morbidity and a prolonged length of hospital stay and place a significant financial burden on health care facilities [3, 4]. Therefore, targeting the recovery of intestinal function after colorectal cancer surgery may contribute to promote rehabilitation for the patient. Now, it is widely accepted that early postoperative feeding is beneficial to decrease the postoperative ileus and shorten the length of hospital stay, and there is significant interest in identifying the measures to stimulate gut function, rather than simply waiting for it to return spontaneously [5–7].

Gum chewing (GC), a kind of sham feeding alternative to early feeding, which is expected to stimulate the cephalic-vagal reflex to increase hormone secretion and then enhance intestinal motility [8], may produce a positive effect on postoperative ileus by reducing postoperative inflammation [9]. However, the underlying mechanisms behind the effects of gum chewing works remain elusive and the results of clinical studies to date are inconclusive, and thus chewing gum has not yet been incorporated into the related guidelines or standard pathways for postoperative nursing [10, 11].

Previous meta-analyses [7, 12–14] have suggested that patients who had gum chewing after surgery might recover intestinal function earlier; however, the sample sizes of the randomized controlled trials (RCTs) included in these analyses were small and an increasing body of relevant research recently have been reported, making it worth reconsidering the evidence on this issue. The aims of this study, therefore, were to review the current evidence on the influence of gum chewing in intestinal function and reassess the efficacy of chewing gum in intestinal function recovery after colorectal surgery.

## 2. Methods

### 2.1. Search Strategy

A general literature search was conducted in PubMed, Embase, Science Direct, and Cochrane library (until April 2017), using the following search terms: “gum-chewing,” “chewing-gum,” “sham-feeding,” “bowel,” “colonic,” “rectal,” “resection,” or “surgery.” We combined these terms in accordance with the instructions of the database. Reference lists and bibliographies from included studies and relevant reviews were used to find additional articles to review. The search was limited to articles written in English, but there was no restriction on publication year.

### 2.2. Study Selection

Two reviewers independently reviewed titles and abstracts to distinguish potentially relevant studies, and articles for more extensive review were selected using the following criteria. Inclusion criteria were as follows: (1) RCT design; (2) study subjects were those who had undergone colorectal cancer surgery; (3) the study made a comparison between gum chewing and standard nursing care post colorectal surgery; and (4) the study contained explicit outcome data. Exclusion criteria were as follows: (1) reviews, systematic reviews, meta-analysis, editorials, case studies, and nonhuman studies; (2) studies not focusing on the effect of chewing gum on colorectal cancer surgery; and (3) studies with no accessible outcome data.

The main outcomes we collected for data analysis included (1) the time to first passage of flatus (days); (2) the time to first defecation (days); (3) the time to first bowel movement (days); (4) the time to start feeding (days); (5) postoperative ileus (POI); (6) postoperative clinical complications such as nausea, vomiting, abdominal distension, and pneumonia; (7) the length of postoperative hospital stay (days); and (8) mortality.

### 2.3. Quality Assessment

The methodological quality and risk of bias of included studies were assessed using the Cochrane Collaboration's “risk of bias” tool, which addresses the seven specific domains: random sequence generation, allocation concealment, blinding of participants and personnel, blinding of outcome assessment, incomplete outcome data, selective reporting, and other sources of bias. Each domain in the tool is achieved by assigning a judgement of “low risk” of bias, “high risk” of bias, or “unclear risk” of bias (Cochrane Handbook for Systematic Reviews of Intervention. Part 2: 8.5) [15].

### 2.4. Data Extraction

Two reviewers independently extracted the following data information: first author, publication year, study design, participants, population, methods of gum chewing, other concurrent interventions, main outcomes, and finding. Disagreements were resolved by discussion or, when necessary, adjudicated by a third reviewer.

### 2.5. Statistical Analysis

Review Manager 5.3 software was used for statistical analyses. We calculated risk ratio (RR) and weighted mean difference (WMD) to present the dichotomous and continuous data, respectively, and 95% confidence interval (CI) was included for all estimates. “Intention to treat” analysis was also conducted. *P* < 0.05 indicated statistical significance. Heterogeneity was tested by applying a chi-squared (*χ*^2^) test and I-squared (*I*^2^) statistic test. A fixed-effect model was adopted when *P* value of *χ*^2^ test > 0.10 and *I*^2^ ≤ 50%, and a random-effect model was adopted otherwise.

## 3. Results

### 3.1. Literature Search

**Figure 1:**
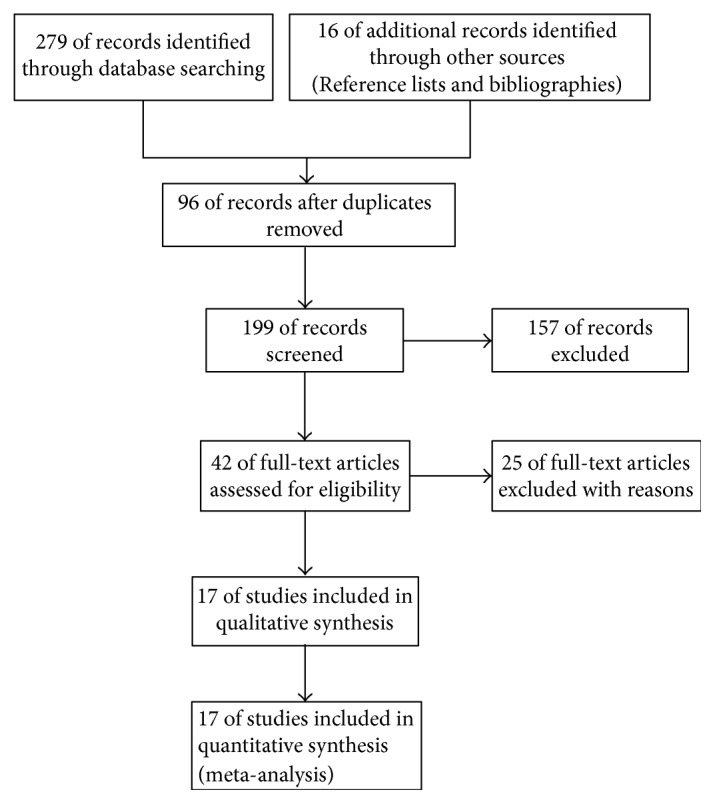
Study flow diagram.

Using the search strategy, 295 potentially relevant references were identified. Of these, 278 articles were excluded due to duplicates or their failure to fulfil predefined inclusion criteria. So finally, we formally included 17 trials with 1845 patients in the main analyses of this review [16–32] (Figure 1).

### 3.2. Study Characteristics

The characteristics of 17 included studies are presented in Table 1. The included studies comprised a total of 1845 patients who had received surgical treatment for colorectal cancer. Of these, 918 followed a gum chewing intervention, and 927 received standard postoperative care. For most studies, gum chewing started from the first day post operation, and the frequency varied from three to four times per day. Although a lot of research confirmed the benefits of chewing gum, the outcomes for the efficacy of gum chewing in each study were still controversial.

### 3.3. Methodological Quality and Risk of Bias

**Figure 2:**
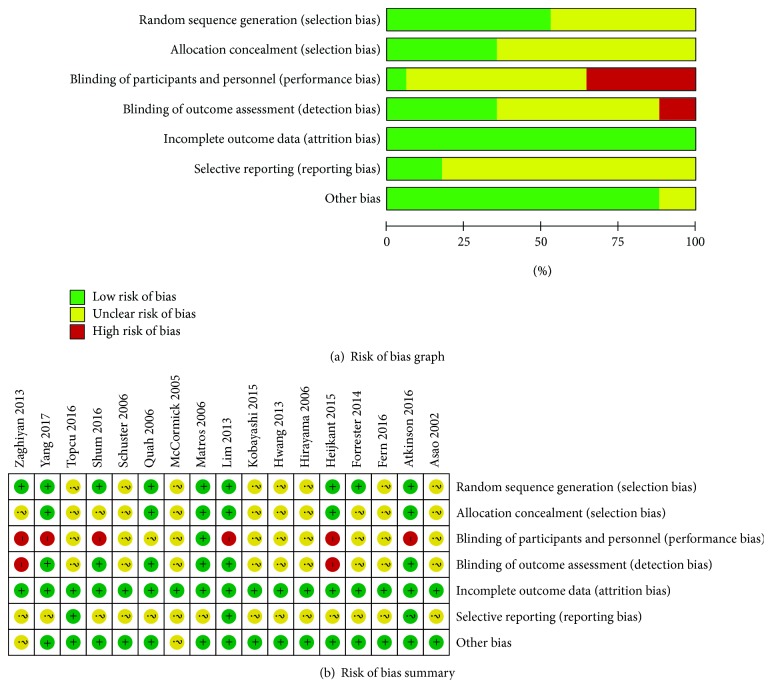
Methodological quality and risk of the included studies.

Figure 2(a) describes all of the bias classifications for the included RCTs, with the summary of qualitative methodological quality according to the bias classification (“low risk,” “unclear risk,” and “high risk”) which was presented in Figure 2(b). Concisely, although all included RCTs mentioned randomization in their research, only nine studies [17, 19, 20, 24, 25, 27, 29, 31, 32] reported correct method of random sequence generation which marked as low risk of selection bias, and only six RCTs [17, 20, 24, 25, 27, 31] described adequate allocation concealments, which serves as a strategy to prevent against ascertainment bias and scored as low risk of bias. In general, sequentially numbered, opaque, sealed envelopes are assigned to each participant to prevent selective bias. Adequate blinding of personnel, participants, and outcome assessment is necessary to prevent against performance and detection bias; however, only one study [25] explicitly described how to maintain patient blinding in all included research, and six RCTs [17, 24, 25, 27, 29, 31] had mentioned the blinding design for assessing the outcomes. Verification on selective reporting of outcomes is necessary because it may help to evaluate the integrity of outcome reporting and protect against bias, yet the majority of the included studies [16, 18–23, 25–29, 31, 32] did not provide sufficient information to assess whether an important risk of bias exists; thus, we judged the risk of other potential sources of bias as unclear. Most of the included studies appear to be free of other sources of bias except for two studies [26, 32], so we judged the risk of bias as low risk for these trials.

### 3.4. Publication Bias Analysis

**Figure 3:**
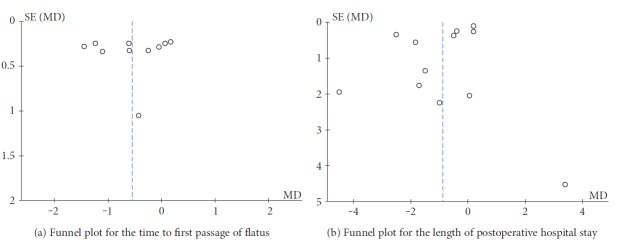


A funnel plot is a simple scatter plot of the intervention effect estimates from individual studies against some measure of each study's size or precision which is used for indication of publication bias [15]. Ten or more studies are needed to enable a funnel plot to give significant evidence of bias, and in this study, we performed a funnel plot analysis on the time to first passage of flatus and the length of postoperative hospital stay. Based on Figures 3(a) and 3(b), we found that the funnel plots were symmetrical, and no significant publication bias was found.

### 3.5. Main Analysis

The effect of gum chewing on multiple parameters of colorectal function following surgery reported in the included studies was assessed. The RRs or WMDs for each study were presented in Figures 4–14.

#### 3.5.1. The Time to First Passage of Flatus

**Figure 4:**
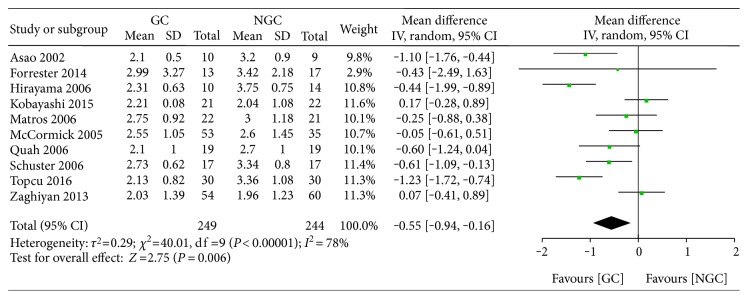
Forest plot for the time to first passage of flatus.

All studies reported the time to the first passage of flatus, yet seven studies [17, 18, 20, 22, 24, 29, 31] failed to provide accurate and necessary data for further analysis. As shown in Figure 4, we found that gum chewing probably shorten the time to first passage of flatus (WMD −0.55; 95% CI −0.94 to −0.16; *P* = 0.006) with a clear statistical heterogeneity across the trials (*I*^2^ = 78%).

#### 3.5.2. The Time to First Defecation

**Figure 5:**
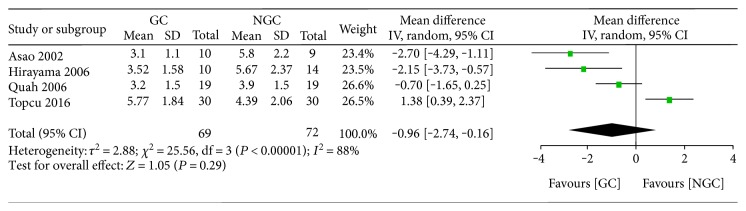
Forest plot for the time to first defecation.

Four studies reported the time to first defecation after colorectal surgery under gum chewing intervention or standard nursing. We found no evidence of a statistically significant difference regarding the time to first defecation between the gum chewing group and control group (WMD −0.96; 95% CI −2.74 to 0.83; *P* = 0.29) (Figure 5), with a clear statistical heterogeneity between trials (*I*^2^ = 88%).

#### 3.5.3. The Time to First Bowel Movement

**Figure 6:**
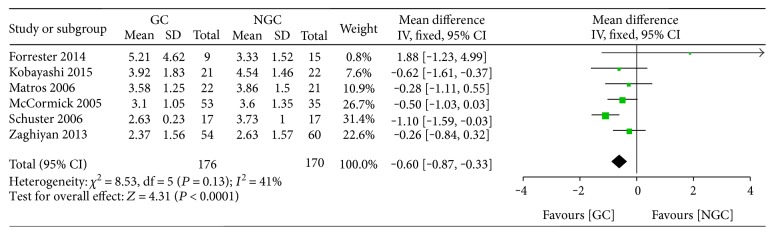
Forest plot for the time to first bowel movement.

Six studies reported the time to first bowel movement; we found that gum chewing shorten the time to first bowel movement compared with the control group (WMD −0.60; 95% CI −0.87 to −0.33; *P* < 0.0001) (Figure 6), with a moderate statistical heterogeneity across the studies (*I*^2^ = 41%).

#### 3.5.4. Time to Start Feeding

**Figure 7:**
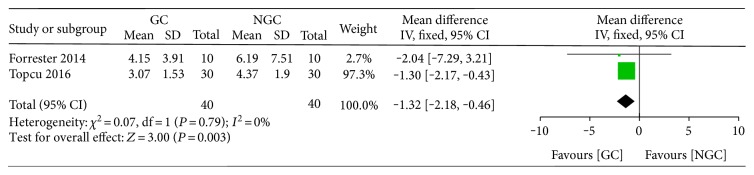
Forest plot for the time to start feeding.

Only two studies reported the time to start feeding; the meta-analysis showed a statistically significant reduction in the time to start feeding for the gum chewing group (WMD −1.32; 95% CI −2.18 to −0.46; *P* = 0.003) (Figure 7), with no evidence of heterogeneity across the trials (*P* value of the homogeneity test = 0.79; *I*^2^ = 0%).

#### 3.5.5. Postoperative Ileus (POI)

**Figure 8:**
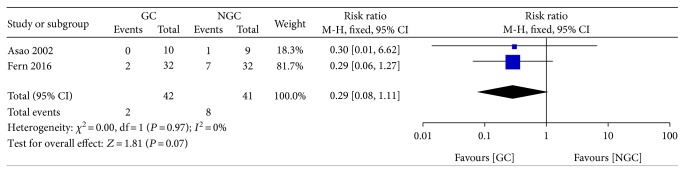
Forest plot for postoperative ileus.

No significant difference was observed between the gum chewing group and control group in the incidence of POI (RR 0.29; 95% CI 0.08 to 1.11; *P* = 0.07) (Figure 8), with no statistical heterogeneity between trials (*P* = 0.97; *I*^2^ = 0%).

#### 3.5.6. Postoperative Nausea

**Figure 9:**
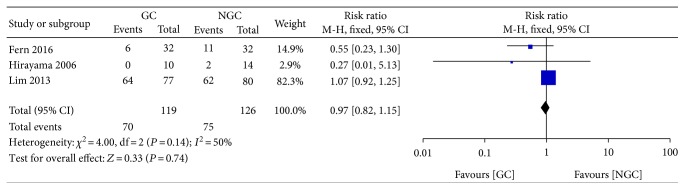
Forest plot for postoperative nausea.

There was no significant difference between the groups in the incidence of postoperative nausea (RR 0.97; 95% CI 0.82 to 1.15; *P* = 0.74) (Figure 9), with a moderate statistical heterogeneity across the trials (*I*^2^ = 50%).

#### 3.5.7. Postoperative Vomiting

**Figure 10:**
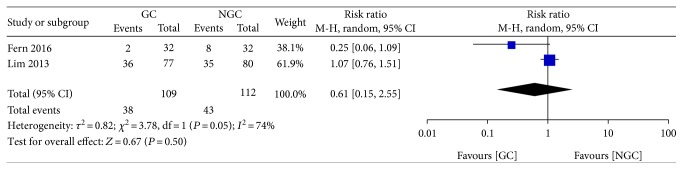
Forest plot for postoperative vomiting.

There was no significant difference between the groups in the incidence of postoperative vomiting (RR 0.61; 95% CI 0.15 to 2.55; *P* = 0.50) (Figure 10), with a clear statistical heterogeneity between trials (*I*^2^ = 74%).

#### 3.5.8. Postoperative Abdominal Distension

**Figure 11:**
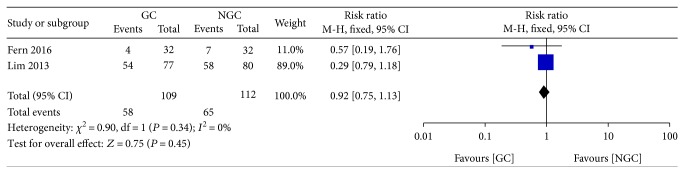
Forest plot for postoperative abdominal distention.

There was no significant difference between the groups in the incidence of postoperative abdominal distension (RR 0.92; 95% CI 0.75 to 1.13; *P* = 0.45) (Figure 11), with no statistical heterogeneity between trials (*P* = 0.34; *I*^2^ = 0%).

#### 3.5.9. Postoperative Pneumonia

**Figure 12:**
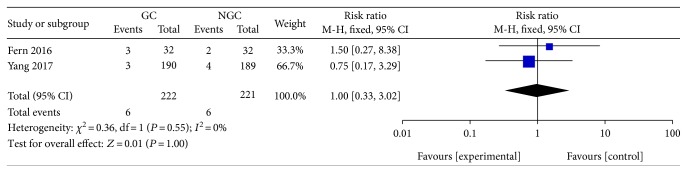
Forest plot for postoperative pneumonia.

No significant difference was observed between the gum chewing group and control group in the incidence of postoperative pneumonia (RR 1.00; 95% CI 0.33 to 3.02; *P* = 1.00) (Figure 12), with no statistical heterogeneity between trials (*P* = 0.55; *I*^2^ = 0%).

#### 3.5.10. The Length of Postoperative Hospital Stay

**Figure 13:**
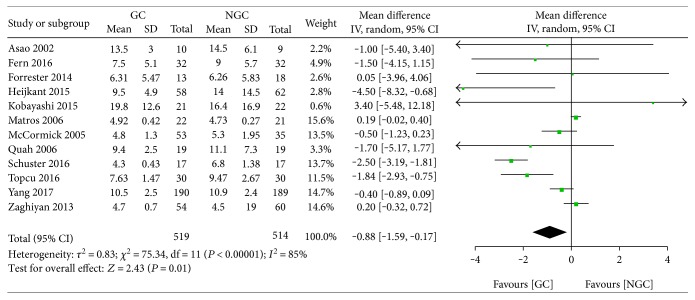
Forest plot for the length postoperative hospital stay.

Twelve studies reported the length of postoperative hospital stay after colorectal surgery under gum chewing intervention or standard nursing. The meta-analysis showed a statistically significant reduction in the length of postoperative hospital stay for the gum chewing group (WMD −0.88; 95% CI −1.59 to −0.17; *P* = 0.01) (Figure 13), with a clear statistical heterogeneity across the studies (*I*^2^ = 85%).

#### 3.5.11. Mortality

**Figure 14:**
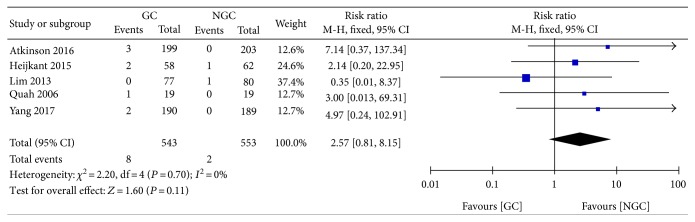
Forest plot for mortality.

There was no significant difference between the groups in the incidence of mortality (RR 2.57; 95% CI 0.81 to 8.15; *P* = 0.11) (Figure 14), with no evidence of heterogeneity across the trials (*P* = 0.70; *I*^2^ = 0%).

### 3.6. Intention to Treat Analysis

**Figure 15:**
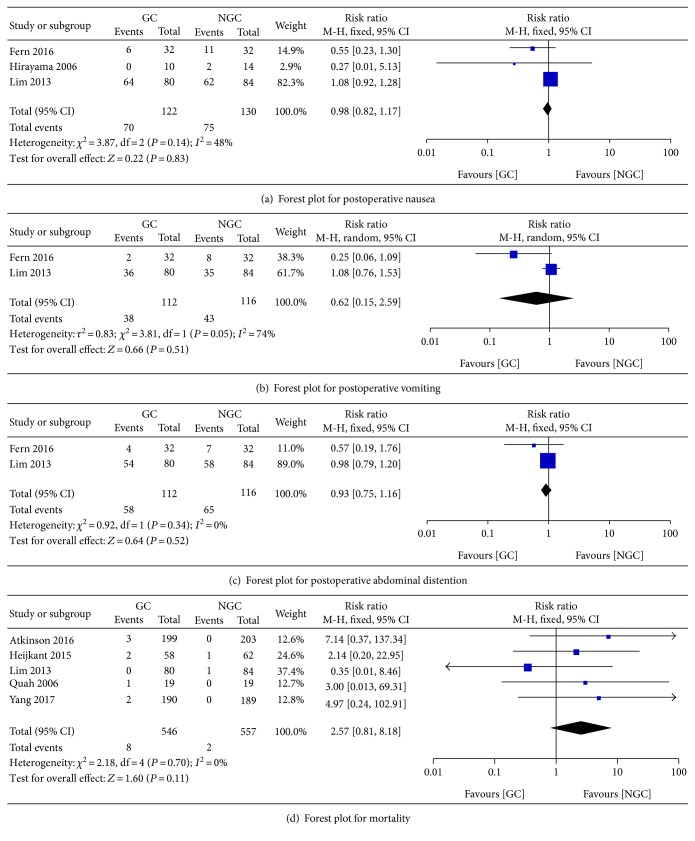
Intention to treat analysis.

In the included studies, ten studies reported the individual withdrawal after randomization. In order to provide unbiased assessments of treatment efficacy, intention to treat analysis was conducted. The result showed that no obvious differences were found in postoperative nausea, vomiting, abdominal distention, and mortality between the gum chewing group and control group (*P* > 0.05), which was consistent with the aforementioned findings (Figure 15).

## 4. Discussion

The results of our meta-analysis have authenticated that chewing gum after colorectal cancer surgery is helpful for the recovery of the patient, which was associated with the reduction of the time to first passage of flatus, first bowel movement, start feeding, and the length of postoperative hospital stay. However, no difference was observed in the incidence of postoperative nausea, vomiting, abdominal distension, pneumonia, and mortality. In the included studies, most of them described the inclusion and exclusion criteria for disease, age, and informed consent, yet only one study [19] mentioned that patients were excluded if they had allergies to chewing gum, and just one research [17] presented the acceptability and compliance of chewing gum, which may result in a risk of selection bias. Additionally, ten studies reported the individual withdrawal after randomization. Remarkably, the main reasons for the patient's withdrawal from the study or not included in the analyses were that patients did not receive chewing gum and inability to chew gum. In order to provide unbiased assessments of treatment efficacy, intention to treat analysis was also conducted in our study. Similarly, we found that there were no obvious differences in postoperative nausea, vomiting, abdominal distention, and mortality between two arms. Complex and various influencing factors of long-term outcome and fewer data were incorporated regarding the outcome of postoperative complications that may account for the phenomenon. In our study, we only included the colorectal surgery but not the whole abdominal surgery (cesarean section, and others) with the following consideration. On the one hand, not all abdominal surgery actually relate to the gastrointestinal tract, and the process of intestinal function recovery may be different following different surgery. Chewing gum acts as sham feeding which, therefore, may promote the recovery of gastrointestinal function through various ways. On the other hand, colorectal surgery changes and affects the integrity of the intestinal tract. The recovery of intestinal function may mainly depend on the process of intestinal tract itself, and chewing gum may provide limited effects on reducing some complications, which just could explain our findings.

How gum chewing works remains unclear. The possible presupposition is that gum chewing mimics the mechanism of food intake, which may significantly stimulate motility in the stomach, duodenum, and rectum [33]; promote gastric, pancreatic, and duodenal secretion [34, 35]; and enhance the release of neuropeptides [33]. The proposed physiologic mechanism is that gum chewing activates the cephalic-vagal axis, which stimulates intestinal myoelectric activity to offset the activation of gastrointestinal opioid receptors [36]. In addition, gum chewing may also stimulate the saliva secretion, resulting in the production of nitrous oxide in sufficient quantities to combat the pathogens in the mouth and gut [37]. Furthermore, gum chewing may offer a better option to regulate a potential risk which is associated with early postoperative enteral or oral feeding.

Recently, a significant amount of research focused on the possible role of chewing gum in the intestinal function recovery, yet the evidence from RCTs remains inconclusive; thereupon, several meta-analyses of the efficacy of gum chewing were published. The meta-analysis by Su'a et al. [12] described the effects of chewing gum on postoperative ileus in adults, showing that chewing gum is beneficial to reducing the time to flatus and the time to bowel motion, but not the length of hospital stay or complications. It is noteworthy that the included patients in this meta-analysis receiving either colorectal surgery or cesarean sections resulted in a heterogeneous patient group. Another meta-analysis [7] of 10 RCTs suggested that sham feeding following colorectal surgery is safe, leading to the improvement in GI recovery, and is associated with the reduction in the length of hospital stay, which is in consistent with our results. Nevertheless, this study found that gum chewing conferred no advantage if patients are placed on a rapid postoperative feeding regime; while our study revealed that gum chewing might accelerate the time to first feeding, the difference may be explained by performing subgroup analysis on whether the rapid postoperative feeding was given to patients, which is just one of the limitations of our research that the corresponding analysis was not implemented. An earlier meta-analysis by Noble et al. [13] identifying nine eligible trials also showed that chewing sugarless gum following elective intestinal resection was associated with improved outcomes, but the efficacy on a reduced rate of clinical complications or reduced cost was not confirmed, which is also in line with our findings. Other previous meta-analyses [38–44] that focused on the effects of gum chewing on the recovery of intestinal function after abdominal surgery (e.g., cesarean delivery) also confirmed the beneficial role of gum chewing after surgery. Currently, several RCTs with moderate or large sample size investigating the efficacy of chewing gum in patients undergoing colorectal resection have been published, however, which has not been included for synthesized analysis in the latest meta-analysis by Song et al. [45]. Consequently, we launch this updated meta-analysis to comprehensively evaluate the effect and safety of chewing gum for patients undergoing colorectal resection, which may have more advantages in reducing heterogeneity and publication bias.

Several limitations of this study should be addressed. Firstly, owing to the differences in patient population and surgery approaches involved, this meta-analysis is limited by the significant heterogeneity of the included studies. Additionally, limited included data subgroup analysis regarding different interventions (e.g., initiation and frequency) in gum chewing was not performed. Besides, there was a lack of blinding in most included studies which may have resulted in significant performance and detection bias. Although blinding participants may be difficult in this setting, blinding observers should be feasible and necessary which is conducive to increasing the reliability of the findings. Therefore, to improve the applicability of study results to individual patients and provide potentially feasible clinical practice guidelines for postoperative nursing, investigators should improve study design to ensure protocol adherence with minimal loss to selection bias risk and follow-up. Moreover, based on the distribution characteristics of prognostic features, how to explore potential causes and search for more powerful evidence still remain to be explored further.

## 5. Conclusions

This meta-analysis indicates that gum chewing in patients undergoing colorectal cancer surgery has a beneficial role in intestinal function recovery. However, the limited data suggests that it offers no benefit in reducing postoperative clinical complications such as nausea, vomiting, abdominal distension, pneumonia, and mortality. Prospective and blinded RCTs are warranted to further clarify the role of chewing gum in the postoperative care in colorectal cancer patients.

## Figures and Tables

**Table 1:** The characteristics of included studies.

Author (year)	Patient population	Number of patients (GC/NGC)	GC intervention	Outcomes	Results
Asao 2002 [16]	Elective laparoscopic colectomy for colorectal cancer	19 (10/9)	Patients chewed gum three times a day from the first postoperative AM until the day they began oral intake.	①②⑤⑦	Gum chewing aids early recovery from postoperative ileus and is an inexpensive and physiologic method for stimulating bowel motility.
Atkinson 2016 [17]	Elective colorectal resection	402 (199/203)	Patients chewed gum for at least 10 min, four times a day for 5 consecutive days (or until discharge, if less than 5 days) from the first postoperative morning.	①③⑥⑦⑧⑩⑪	Chewing gum did not alter the return of bowel function or LOS after colorectal resection.
Fernandez 2016 [18]	Elective colorectal surgery	64 (32/32)	Chewing gum within the first 24 h after surgery, for 15 minutes every four hours throughout their hospital stay, with six resting hours at night.	①⑤⑥⑦⑧	The use of chewing gum after colorectal surgery was associated with less postoperative ileus and vomiting, and with an increased passage of flatus within the first 48 hours after surgery.
Forrester 2014 [19]	Open colon resection or a laparoscopic sigmoid colectomy	31 (13/18)	Gum chewing began on the morning of the first postoperative day or after removal of the nasogastric tube, and the times of gum chewing corresponded with routine medication administration times.	①③④⑦⑨	Postoperative gum chewing was not more effective than standard postoperative care or attention-control intervention in reducing the duration of postoperative ileus symptoms, length of stay, or complications among patients following open laparoscopic sigmoid colectomy.
Heijkant 2015 [20]	Elective colorectal surgery	120 (58/62)	Patients started chewing gum 3 h before and after surgery. The frequency and duration of gum chewing were not standardized.	①②⑤⑦⑧⑫	Gum chewing is a safe and simple treatment to reduce POI and is associated with a reduction in systemic inflammatory markers and complications.
Hirayama 2006 [21]	Elective open surgery for colorectal cancer	24 (10/14)	Patients chewed gum three times daily for 30 minutes every time.	①②⑥	Gum-chewing provides a simple and effective method to improve the postoperative state of patients.
Hwang 2013 [22]	Laparoscopic colorectal cancer surgery	132 (65/67)	Patients started to chew gum on the first postoperative day, 3 times a day, approximately 10–20 minutes at a time, until normal feeding was resumed.	①⑦	Gum chewing is an easy and cost-effective method to reduce the length of the postoperative hospital stay for laparoscopic colorectal cancer surgery.
Kobayashi 2015 [23]	Left-sided resection for colorectal cancer	43 (21/22)	Patients chewed gum three times daily for ≥5 minutes (morning, noon, and evening) until the first day of regular oral dietary intake.	①③⑦⑬	Gum chewing changed the serum levels of des-acyl ghrelin and gastrin, but we were unable to demonstrate an effect on the recovery of bowel function.
Lim 2013 [24]	Colorectal resection	157 (77/80)	Chewing gum for 15 minutes 4 times a day (8 AM, 12 PM,4 PM, and 8 PM).	①③⑥⑦⑧	Chewing gum is safe, but does not hasten the return of gastrointestinal function in patients who receive accelerated postoperative feeding.
Matros 2006 [25]	Elective partial colectomy for colorectal cancer	43 (22/21)	Chewing gum for 45 minutes three times daily at 9 AM, 4 PM, and 8 PM.	①③⑦	Gum chewing, although safe, does not reduce duration of post colectomy ileus.
McCormick 2005 [26]	Elective colon resection	88 (53/35)	Gum chewing 4 times a day for 15 min.	①③⑦	Chewing gum in the postoperative period may be an inexpensive intervention to facilitate recovery and decrease hospital cost.
Quah 2006 [27]	Open surgery for left-sided colorectal cancer	38 (19/19)	The patients chewed gum for at least 5 min, three times daily from the first postoperative morning until the intake of a solid diet orally.	①②⑦⑧⑭	The addition of gum chewing to a standardized postoperative regimen did not reduce the period of postoperative ileus or shorten length of stay following open surgery for left-sided colorectal cancer.
Schuster 2006 [28]	Elective open sigmoid resections	34 (17/17)	Chewing gum 3 times daily for 1 hour each time until discharge.	①③⑦⑨	Gum chewing speeds recovery after elective open sigmoid resection by stimulating bowel motility.
Shum 2016 [29]	Laparoscopic colorectal resection	82 (41/41)	Chewing gum three times daily for 30 min each time from day 1 until discharge.	①③⑦⑨	Chewing gum is a simple intervention that speeds intestinal transit in patients managed with a recovery programme after laparoscopic colorectal resection
Topcu 2016 [30]	Planned open colorectal surgery	60 (30/30)	Patients chewed gum three times a day after meals for a period of 15 min from the first morning after the operation until the time of their discharge.	①②④⑦	Chewing gum is a simple intervention for reducing postoperative ileus after colorectal surgery.
Yang 2017 [31]	Open or laparoscopic resection	394 (197/197)	Chewing gum three times daily for at least 10 min, starting on postoperative day 1 and continuing for 5 consecutive days or until flatus.	①③⑥⑦⑧	Chewing gum does not appear to affect recovery of bowel function or hospital stay, though it may benefit patients who undergo open resection.
Zaghiyan 2013 [32]	Major colorectal surgery	114 (54/60)	Chewing gum for 45 minutes, 3 times daily on postoperative days 1 to 7.	①③⑥⑦	There does not appear to be any benefit to sugared chewing gum in comparison with no gum in patients undergoing major colorectal surgery

Note: ① the time to first passage of flatus; ② the time to first defecation; ③ the time to first bowel movement; ④ time to start feeding; ⑤ postoperative ileus (POI); ⑥ clinical complications such as nausea, vomiting, abdominal distension, pain, pneumonia, wound infection, bleeding, and others; ⑦ the length of postoperative hospital stay; ⑧ mortality; ⑨ time to first hunger; ⑩ first day of auscultated bowel sounds; ⑪ solid food consumption and tolerance; ⑫ local and systemic inflammation and gastric emptying; ⑬ gut hormones; ⑭ tolerance and satisfaction of gum chewing.
